# Nanocarrier-Based Delivery Systems for Natural Compounds Across Research Stages

**DOI:** 10.3390/ma18214960

**Published:** 2025-10-30

**Authors:** Antonella Antonelli, Francesco Palma

**Affiliations:** Department of Biomolecular Sciences, University of Urbino Carlo Bo, Via Cà Le Suore 2/4, 61029 Urbino, PU, Italy; antonella.antonelli@uniurb.it

**Keywords:** natural compounds, nanocarriers, drug delivery, polymeric nanoparticles, targeting, preclinical applications, clinical translation, RBC-based delivery systems

## Abstract

Natural compounds such as polyphenols, flavonoids, and terpenoids have long been explored for their therapeutic potential. They can act as antioxidants, limit inflammation, and influence cancer or neurodegenerative pathways. However, these benefits rarely translate directly into medical practice, as their solubility is poor, chemical stability is fragile, and metabolism is too fast. In recent years, nanotechnology has offered an alternative route. A wide range of materials, polymeric, inorganic, hybrid, or responsive to external stimuli, were designed to protect and deliver such molecules. Each platform features different preparation methods and release behaviors; all intended to extend circulation and increase tissue selectivity. Considerable attention was paid to targeting strategies, both passive and ligand-mediated, that enhance accumulation in diseased tissues. Preclinical studies have confirmed that encapsulation can raise the therapeutic index of phytochemicals against various conditions, including cancer, inflammation, microbial infections, and neurodegeneration. Still, translation to the clinic is far from resolved, limited by uncertainties over safety, manufacturing scale, and regulation. A parallel line of research now investigates biomimetic carriers, including vesicles derived from red blood cells and whole erythrocytes, which offer immune evasion and versatile loading capacity. The convergence of nanotechnology and natural product pharmacology, enriched by such biologically inspired designs, may open the way to more precise, multifunctional, and patient-tailored therapies.

## 1. Introduction

Natural bioactive compounds, including polyphenols, flavonoids, alkaloids, and terpenes, have historically played a central role in drug development, with over 60% of approved small-molecule drugs are directly or indirectly derived from natural sources [1,2]. These phytochemicals have been extensively studied for their pharmacological properties, which include antioxidant, anti-inflammatory, anticancer, neuroprotective, and antimicrobial activities, with significant potential in the prevention and treatment of complex diseases such as cancer, neurodegenerative disorders, cardiovascular diseases, and microbial infections [1,2,3].

However, their clinical translation is strongly limited by unfavorable physicochemical and pharmacokinetic characteristics: low water solubility, instability under physiological conditions, rapid metabolism, poor oral bioavailability, and suboptimal systemic distribution profiles [3,4]. Curcumin-loaded PLGA and PLGA-PEG nanoparticles significantly increased plasma exposure and blood residence time after oral administration in rats [3,4]. Furthermore, PLGA-PEG-PLGA triblock copolymer micelles further prolonged the terminal half-life and mean residence time by approximately 4.5- and 2.7-fold, respectively, redistributing the biodistribution toward lung and brain and reducing hepatic and splenic uptake [5].

Over the past two decades, nanotechnology applied to drug delivery has provided innovative tools to address these issues, enabling improvements in stability, solubility, absorption, and tissue targeting [5,6]. Due to their small size and high surface-to-volume ratio, nanoparticles can encapsulate phytochemicals, protecting them from degradation.

**Figure 1:**
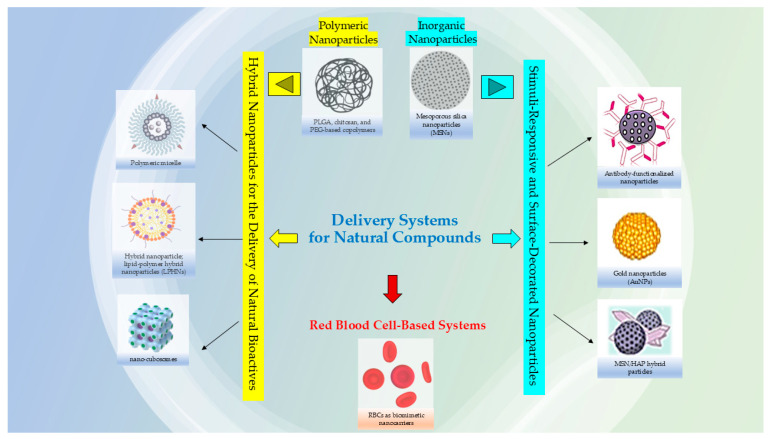
Examples of nanocarriers for natural bioactive compounds.

Researchers are also considering the utility to using nanocarriers for complex plant extracts, rather than only isolated compounds; this can be advantageous to reduce the synthesis of chemical precursors and potentially lower costs but in the meantime still harnessing the combined therapeutic properties of the extract and the nanocarrier that could enhance the bioactivity due to synergistic effects that amplify desired actions, such as antimicrobial or antioxidant properties; the mixture of compounds in an extract can work together synergistically, enhancing the overall therapeutic effect compared to single, isolated compounds [7,8]. The use of both plant extracts and nanocarriers can potentially offer a broader range of therapeutic effects compared to a single isolated metabolite, and in some cases, minimize the toxicity associated with the materials used in the nanosystems [9,10]. Moreover, loading an entire extract into nanocarrier systems might also improve stability by protecting it from degradation, and nanocarriers (Figure 1) can be engineered to target specific tissues and control the release of the active ingredients [11,12].

This approach enables the development of more effective, potent, and stable treatments with fewer side effects. It is applicable in various areas, including drug delivery, where nanocarriers can improve the therapeutic effects of natural products like alkaloids and terpenoids, which often have poor solubility [13,14]. These nanocarriers also impact their release profiles and help target accumulation in diseased tissues through passive strategies, such as enhanced permeability and retention (EPR), and active strategies, like surface modification with specific ligands [15,16,17,18].

In addition, the development of multifunctional and stimuli-responsive nanocarriers is opening new possibilities for controlled release and the combination of therapeutic and diagnostic functions (theranostic approach) [17,19].

Despite these advances, the literature still lacks systematic analyses that critically connect material design (polymeric, inorganic, hybrid, stimuli-responsive), formulation techniques, targeting strategies, and pharmacodynamic (PD) and pharmacokinetic (PK) outcomes obtained in vivo, with a specific focus on clinical translation barriers and regulatory aspects [13,20,21]. Many published reviews focus on synthetic drugs or provide general overviews of phytopharmaceutical formulations, but few examine in depth the nanocarriers specifically engineered for the delivery of natural compounds, analyzing both preclinical performance and prospects for clinical application [2,6,13].

In addition to synthetic nanocarriers, biomimetic platforms such as red blood cell (RBC)-derived vesicles are gaining increasing attention. By exploiting natural membrane components, these systems provide intrinsic biocompatibility, immune evasion, and prolonged circulation, representing a promising complement to polymeric, inorganic, and hybrid formulations for the delivery of natural compounds [22,23].

This review aims to fill this gap by providing a critical and detailed overview of the main nanomaterials developed for the delivery of natural bioactive compounds, organized according to carrier composition (polymeric, inorganic, hybrid, stimuli-responsive), targeting mechanisms, and release modalities. The specific objectives are:

- to classify and compare the main types of nanocarriers used for phytochemical delivery;
- to describe in detail the formulation techniques, encapsulation efficiencies, and release profiles achieved;
- to analyze tissue-targeting strategies, both passive and active, and their impact on selective drug accumulation;
- to summarize the most promising preclinical results, with a focus on pharmacodynamic and pharmacokinetic outcomes in cancer, inflammation, neurodegeneration, and infection models;
- to discuss the main barriers to clinical translation, including regulatory issues, long-term safety, and production scalability;
- to propose perspectives for future development in light of emerging trends and unmet clinical needs.

Through this integrated analysis, the review aims to provide researchers, clinicians, and pharmaceutical developers with a useful resource to guide the design of next-generation nanodelivery systems that can enhance the therapeutic potential of natural compounds and facilitate their transition from laboratory research to clinical practice.

## 2. Trends in Curcumin Research and the Emerging Role of Nanoparticle-Based Delivery

To better understand the evolution of curcumin research in the biomedical field, a targeted literature analysis was conducted using the PubMed database through the NCBI Entrez E-utilities API. The search strategy focused on original research articles published between 2020 and 2025, written in English, and explicitly mentioning “curcumin” (or synonyms) in the title or abstract. Review articles were excluded. To ensure biomedical relevance, queries included at least one of the following disease-related keywords in Title/Abstract: “disease”, “cancer”, “tumor”, “tumour”, or “neoplasm”. Subsets were defined on the basis of research context: in vitro (absence of animal-related keywords, excluding clinical trial filters), animal studies (including “mouse”, “mice”, “rats”, “feline”, “canine”, or “in vivo” in Title/Abstract), and clinical trials (filtered by “Clinical Trial” [Publication Type]). Within each category, publications were further classified according to the presence of nanoparticle-related terms such as “nanoparticle”, “nanocarrier”, “liposome”, “micelle”, “nanoemulsion”, “nanogel”, “solid lipid nanoparticle”, “nanostructured lipid carrier”, “nanocrystal”, “dendrimer”, “nanosphere”, “nanocapsule”, “nanocomposite”, or “nanocurcumin”.

**Figure 2:**
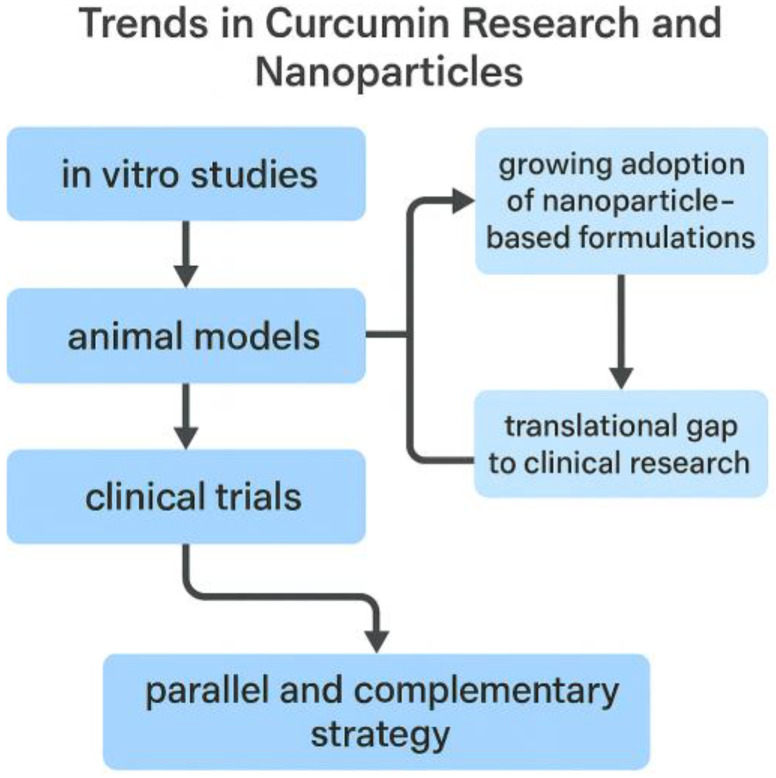
Conceptual overview of publication trends on curcumin research in the biomedical field (2020–2025). The analysis highlights the progressive integration of nanoparticle-based delivery systems across research stages (in vitro, preclinical animal models, and clinical trials), with increasing but still limited clinical translation.

This classification is conceptually summarized in Figure 2, which illustrates the progressive integration of nanoparticle-based delivery systems across the three stages of curcumin research (in vitro, animal, and clinical) and highlights the translational challenges that still need to be overcome.

The Supplementary Materials (File S1, pubmed_trends.py) provide the complete PubMed queries together with the Python 3.12 script used for data retrieval and classification. This resource ensures reproducibility of the bibliometric analysis described in this section.

Between 2020 and 2025, curcumin research remained dominated by in vitro assays, followed by animal studies, while clinical trials accounted for only a small fraction of the output. Around one-third of laboratory and preclinical studies involved nanoformulations, with values generally ranging from 27% to 37%. Clinical adoption was much less consistent, oscillating between 9% and 20% in most years. For 2025, only one nanoparticle-based trial was retrieved up to the present, corresponding to 7% of the clinical subset. This pattern confirms the translational bottleneck: nanotechnology is widely explored in proof-of-concept settings but is rarely advanced to patient studies [13,24,25].

**Figure 3:**
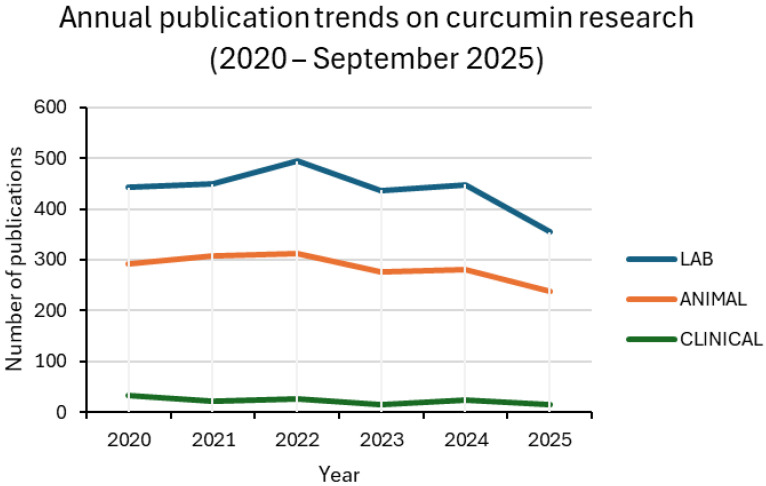
Annual publication trends on curcumin research in the biomedical field (2020 –2025). The line plots show the number of original research articles in three categories: in vitro studies (LAB), preclinical animal studies (ANIMAL), and clinical trials (CLINICAL). Each category includes both conventional and nanocarrier-based studies. Data were retrieved from PubMed using NCBI Entrez E-utilities with the search terms specified in Section 2.

**Figure 4:**
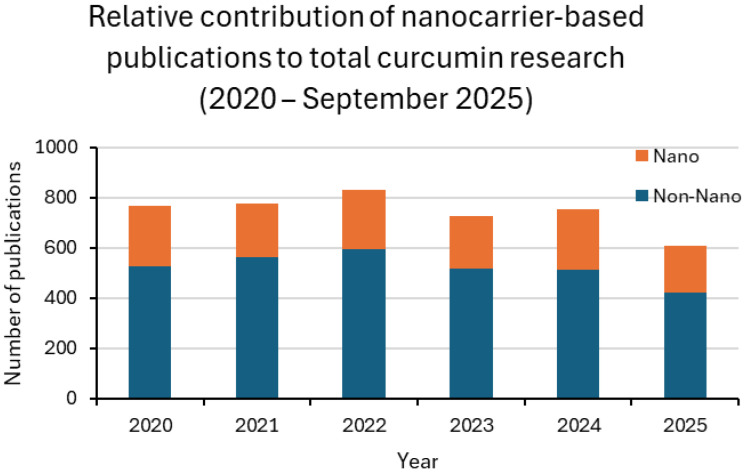
Relative contribution of nanocarrier-based publications to total curcumin research (2020–2025). Stacked bar plots display the proportion of studies explicitly mentioning nanoparticle-related terms (Nano, blue) compared with conventional approaches (Non-Nano, orange). The analysis highlights the greater integration of nanotechnology in preclinical research compared with the clinical setting, where nanoparticle-based trials remain scarce.

Taken together, these findings indicate that nanocarrier development is advancing in parallel with conventional approaches rather than replacing them. Nanotechnology is increasingly applied in preclinical research to enhance solubility, stability, and bioavailability, but its clinical translation still faces significant barriers related to safety, scalability, and regulatory approval [24,25]. Figure 3 and Figure 4, together with Table 1, summarize these results, highlighting both the growing integration of nanotechnology across experimental settings and the persistent translational gap that must be overcome.

## 3. Advanced Nanomaterials for the Delivery of Natural Bioactives

The use of nanomaterials as delivery systems for natural bioactive compounds has attracted considerable attention in the last decade due to their ability to overcome the major pharmacokinetic and pharmacodynamic limitations of these molecules. Many natural compounds, including polyphenols, flavonoids, terpenoids, and alkaloids, exhibit poor water solubility, chemical instability, rapid metabolism, and low bioavailability, which restricts their therapeutic potential [2,3,6].

Nanotechnology-based carriers can improve solubility, protect bioactives from degradation, enhance absorption across biological barriers, prolong systemic circulation, and provide site-specific delivery, ultimately amplifying pharmacological effects while reducing systemic toxicity [3,16,26]. Furthermore, the nanoscale size of these carriers allows for passive accumulation in inflamed or tumor tissues through the enhanced permeability and retention (EPR) effect, while surface functionalization can be employed for active targeting of specific receptors [15,27].

**Figure 5:**
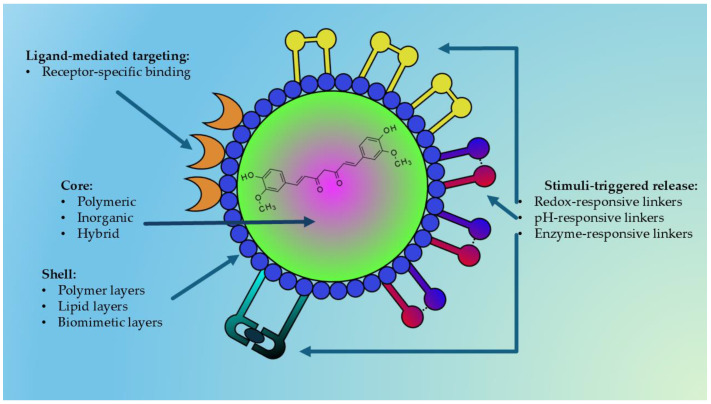
Schematic representation of a nanoparticle designed for the delivery of natural bioactive compounds (e.g., curcumin). The structure consists of an inner core—polymeric, inorganic, or hybrid—selected according to the physicochemical characteristics of the encapsulated compound or extract. The core is surrounded by a polymeric, lipidic, or biomimetic shell that provides structural stability, stealth properties, and biological compatibility. Surface ligands enable active targeting toward specific receptors, while stimulus-responsive linkers mediate controlled drug release triggered by pH variations, intracellular glutathione (GSH), or enzymatic activity. This modular architecture allows fine regulation of pharmacokinetics and site-selective release of natural compounds.

Among the various nanocarriers investigated, polymeric, inorganic, hybrid, and stimuli-responsive nanoparticles are particularly promising due to their versatility, tunable physicochemical properties, and potential for multifunctionality. Figure 5 represents a general structure of a multifunctional nanoparticle used for delivering natural bioactive compounds.

This chapter discusses recent advances in these four major classes of nanomaterials, focusing on composition, fabrication techniques, encapsulation strategies, biological performance, and translational prospects for the delivery of natural bioactives.

### 3.1. Polymeric Nanoparticles for the Delivery of Natural Bioactives

Polymeric nanoparticles (NPs) represent one of the most widely studied classes of nanocarriers for the delivery of natural compounds that are poorly soluble or chemically unstable. Among them, biodegradable polymers such as poly(lactic-co-glycolic acid) (PLGA), chitosan, and polyethylene glycol (PEG)-based copolymers are particularly attractive, as they can improve the solubility, stability, and bioavailability of polyphenols, flavonoids, and other phytochemicals [2,3,4,5,28]. Beyond mucosal delivery, chitosan derivatives also find applications in tissue repair, as shown by Tiboni et al. (2022), who combined chitosan with sugar esters to promote wound healing in vivo [29].

The intrinsic biocompatibility of these polymers, combined with the possibility of adjusting their degradation rate through formulation choices, expands their range of applications. Another advantage lies in their ability to incorporate both hydrophilic and hydrophobic molecules, which makes them suitable for multiple administration routes, including oral, parenteral, and even intranasal delivery [3,4,15].

Attention has also turned to how structural design affects biological behavior. One illustrative case is melting electrospinning writing (MEW), an additive manufacturing approach that enables the fabrication of microfibers with controlled pore size and fiber orientation at the micrometer scale. In their work, Eichholz and Hoey showed that altering the alignment of polycaprolactone (PCL) fibers, even by small degrees (90°, 45°, 10°, or random), alters porosity and mechanical stiffness, creating microenvironments that can direct stem cell growth and differentiation [26].

Based on these structural innovations, it is equally important to consider the choice of polymers and formulation techniques that determine the performance of nanoparticles.

Among the available polymers, PLGA remains by far the most widely used for encapsulating hydrophobic nutraceuticals such as curcumin and resveratrol, thanks to its FDA approval, biodegradability, and favorable safety profile.

Building on these structural innovations, it is equally important to consider the choice of polymers and the formulation techniques that dictate nanoparticle performance.

In particular, curcumin-loaded PLGA and PLGA-PEG nanoparticles were shown to significantly improve pharmacokinetic parameters after oral administration [3], while PLGA-PEG-PLGA-based triblock copolymer micelles provided prolonged half-life and improved biodistribution to the brain and lungs after intravenous administration [4].

The introduction of PEGylation has further improved these systems by extending circulation times and improving tissue distribution, mainly through reduced opsonization and slower clearance by the reticuloendothelial system [3]. In addition to PLGA, other polymers, such as PEG-PBLG, Eudragit, and chitosan derivatives, were also explored to promote mucoadhesion and improve absorption across biological barriers [15].

A variety of formulation techniques were optimized to achieve these results. For example, solvent evaporation in emulsion, in its simple or dual form, is often employed to encapsulate both hydrophobic and amphiphilic compounds [3,4]. Alternatively, nanoprecipitation or solvent-displacement methods enable the production of nanoparticles with narrow size distributions, while the self-assembly of amphiphilic block copolymers, such as PLGA-PEG-PLGA micelles, enables high encapsulation efficiency and controlled release of active natural molecules [5,15].

Most often, the resulting nanoparticles exhibit diameters between 100 and 300 nm, surface charges (zeta potential) optimized for colloidal stability, and drug-loading efficiencies often exceeding 60–80%, depending on specific formulation parameters [3,4,5].

Importantly, the adoption of these formulation approaches was repeatedly linked to significant pharmacokinetic improvements in preclinical models.

The encapsulation of natural bioactive molecules within polymeric nanoparticles was consistently shown to enhance their pharmacokinetic performance, particularly in terms of oral bioavailability and systemic exposure. A notable example is provided by Khalil et al., who demonstrated that curcumin-loaded PLGA and PLGA-PEG nanoparticles increased the maximum plasma concentration (C_max_) by 2.9- to 7.4-fold and improved overall bioavailability by up to 55-fold compared to a free curcumin suspension in rats [3]. In agreement with these findings, Xie et al. reported that PLGA-encapsulated curcumin showed superior absorption and significantly prolonged half-life in vivo [4].

Micellar systems further extend these advantages. PLGA-PEG-PLGA-based formulations not only sustained curcumin release for several hours but also significantly modified its pharmacokinetic profile, favoring its distribution to specific tissues in preclinical models [5]. By reducing rapid metabolism and improving gastrointestinal fluid stability, these nanosystems provide a robust strategy to overcome the inherent limitations of free curcumin and similar natural compounds.

In addition to improving systemic pharmacokinetics, polymeric nanocarriers have also been engineered to exploit alternative delivery routes and targeting strategies.

One of the most innovative approaches explored in recent years is the intranasal administration of PEG-PLGA nanoparticles conjugated with odoranalectin, a lectin able to selectively bind the olfactory epithelium. This strategy allowed the direct administration of curcumin to the central nervous system (CNS), effectively bypassing first-pass metabolism and significantly improving brain bioavailability [15].

Building on these results, further studies have confirmed the potential of intranasal PLGA nanoparticles to exploit the olfactory and trigeminal pathways as natural entry routes into the CNS. This administration mode is particularly appealing for natural compounds with neuroprotective properties, which are otherwise limited by rapid metabolism and low systemic bioavailability. To optimize their performance, PLGA-based systems were engineered with mucoadhesive coatings or PEGylation, thereby prolonging nasal residence time, enhancing epithelial permeation, and facilitating efficient nose-to-brain transport [30,31]. Notably, both curcumin- and resveratrol-loaded PLGA nanoparticles have demonstrated superior pharmacokinetics and enhanced distribution within the brain following intranasal delivery, underscoring their therapeutic promise in neurodegenerative disorders [15,30,31]. These findings highlight how the interplay between nanocarrier design and administration route is crucial for maximizing the therapeutic index of phytochemicals. In this context, ligand-modified polymeric systems exemplify the potential of active targeting to achieve selective tissue delivery.

While polymeric nanocarriers provide multiple advantages, critical limitations, ranging from scalability to regulatory hurdles, must still be addressed.

Polymeric nanoparticles offer several advantages that make them particularly interesting for the delivery of natural bioactive substances. They can encapsulate significant quantities of drug molecules while protecting them from chemical or enzymatic degradation and provide controlled, sustained release profiles that help maintain therapeutic concentrations over time. Furthermore, their surfaces can be easily functionalized, allowing both passive targeting via physicochemical regulation and active targeting via the conjugation of ligands or antibodies.

Although they recognized biocompatibility, both chitosan and PEG-based polymers also present intrinsic pharmacological limitations. Native chitosan shows poor solubility at physiological pH and variable deacetylation degrees, affecting reproducibility and drug–polymer compatibility; while PEG coatings may elicit anti-PEG antibodies and hypersensitivity reactions upon repeated administration. These immune responses, associated with the accelerated blood clearance (ABC) phenomenon and limited PEG biodegradability, can compromise long-term efficacy and cause hepatic or lysosomal accumulation [32,33,34,35]. Accordingly, recent efforts have focused on chitosan derivatization and on the exploration of alternative stealth polymers such as polysarcosine (pSar) or poly(hydroxypropyl methacrylamide) (PHPMA) to minimize immunogenicity while preserving circulation stability. Moreover, since the adsorption of plasma proteins (protein corona) can markedly alter nanoparticle behavior, studies have shown that coatings based on PEG, polysarcosine, or PHPMA reduce nonspecific protein binding and help maintain predictable pharmacokinetics, thus mitigating patient-to-patient variability observed with PEGylated carriers [33,36,37].

While these strengths are significant, limitations remain. Large-scale production remains a challenge, as variability in encapsulation efficiency and the generation of polymer degradation byproducts can impact reproducibility. Furthermore, for some natural compounds, the drug-loading capacity is relatively low, limiting their clinical applicability. Beyond these technical aspects, regulatory hurdles also persist, particularly regarding the demonstration of long-term safety and batch-to-batch production standardization, both essential requirements for successful clinical translation.

Nevertheless, ongoing advances in polymer functionalization, stimuli-responsive micelles, and nose-to-brain systems suggest that polymeric nanoparticles will continue to be a cornerstone of nanomedicine strategies for natural bioactive compounds.

### 3.2. Inorganic Nanoparticles for the Delivery of Natural Bioactives

Inorganic nanoparticles (NPs) have recently attracted attention as highly versatile platforms for delivering natural bioactive compounds. Their distinctive characteristics, including structural stability, large surface area, and adjustable porosity, make them particularly suitable for accommodating and protecting delicate phytochemicals. Furthermore, the possibility of multifunctionalization allows these carriers to combine therapeutic, diagnostic, and targeting roles within a single system, an approach often referred to as theranostics [6,16,17,18]. Several inorganic materials have already shown great promise in this context.

**Figure 6:**
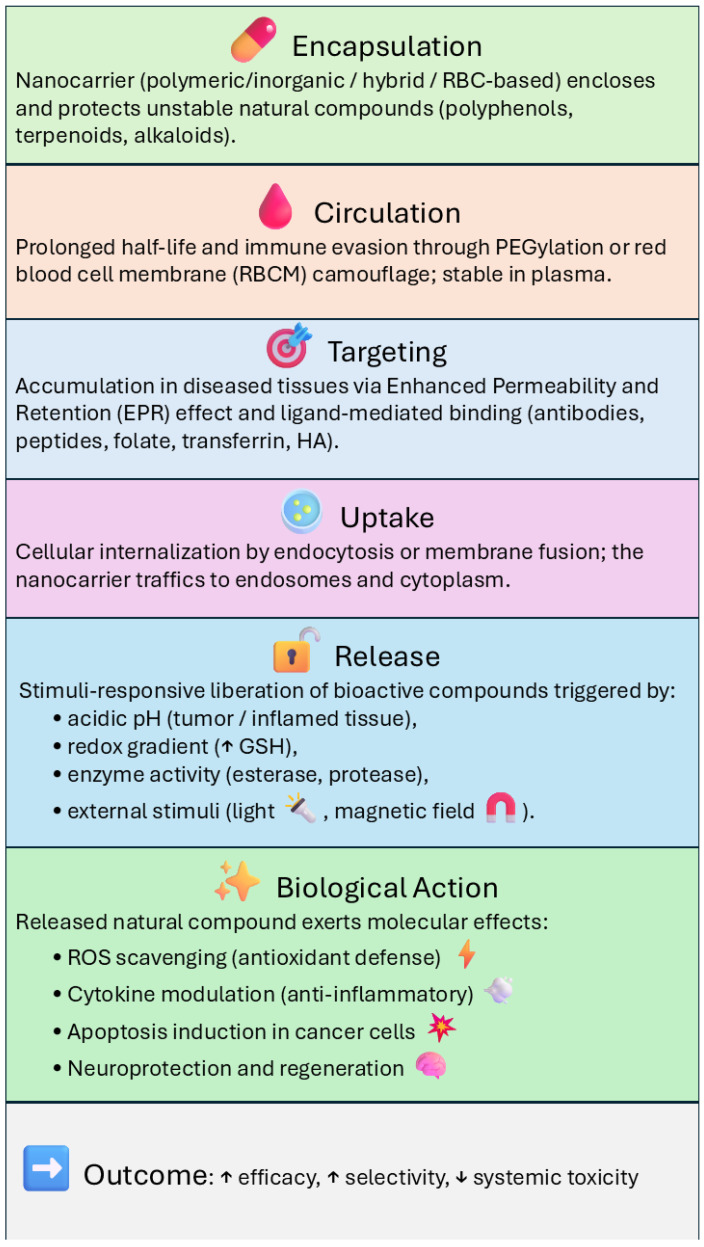
Diagram of the mechanisms of biological activity of different types of nanocarrier-delivery systems. Upward (↑) and downward (↓) arrows indicate an increase or a decrease of the corresponding parameter, respectively.

In addition to polymeric nanocarriers, inorganic systems such as mesoporous silica nanoparticles (MSNs), gold nanoparticles (AuNPs), and magnetic or hybrid scaffolds have also been explored for the delivery of natural compounds. As discussed in recent reviews [13], these platforms can enhance drug loading, regulate release kinetics, and help preserve the biological activity (Figure 6) of sensitive phytochemicals, including quercetin, epigallocatechin gallate (EGCG), and curcumin. Within this group, MSNs stand out because their ordered porous structure and large surface area provide particularly high loading capacity for poorly soluble molecules.

Mesoporous silica nanoparticles (MSNs) are characterized by their ordered pore structures, typically between 2 and 50 nm, combined with large surface area and tunable pore volumes. These characteristics make them particularly effective for loading poorly soluble phytochemicals, ensuring high drug incorporation and stability [6,18].

Recent studies clarified the molecular basis linking pore size to drug loading and release efficiency in mesoporous silica nanoparticles (MSNs). Dendritic MSNs with intermediate pores (~15 nm) demonstrated the highest loading efficiency, due to a balanced combination of diffusion and adsorption at silanol sites [38]. Additionally, research revealed a pore-size-dependent relationship between drug loading and release kinetics, with small pores (3–5 nm) promoting molecular confinement and hydrogen-bonding stabilization, and larger pores (>10 nm) facilitating solvent accessibility and faster release [39]. These findings, together with the review by Benkő et al. (2025), highlight that the molecular interactions between small hydrophobic molecules and the silica surface, mainly through hydrogen bonding and physical confinement within mesopores, govern both the amorphization and stability of the encapsulated compound [40].

Further innovations have focused on improving the biological performance of MSNs through surface modifications [41]. In fact, recent studies have demonstrated that the amine functionalization of MSN results in a more favorable release profile and increased bioavailability of curcumin compared to free curcumin. Additionally, this study highlights the promising features of amine-functionalized MSN as a carrier for delivering low-solubility drugs [42].

In a notable example, Sapino et al. [6] demonstrated that quercetin-loaded MSNs not only improved the chemical stability of the compound but also provided controlled release, resulting in superior dermal permeation compared to free quercetin solutions [6].

Amino-functionalization or hydroxyapatite (HAP) coating was shown to significantly improve their biocompatibility, also imparting specific antibacterial properties. Yi et al. [18] reported that such hybrid MSNs achieved drug loadings of approximately 28–30% and sustained release over several hours, clearly outperforming unmodified formulations in antimicrobial testing [18]. These results illustrate how fine-tuning the surface chemistry of MSNs can expand their therapeutic potential beyond simple drug delivery.

Gold nanoparticles (AuNPs) have also emerged as valuable carriers, combining biocompatibility with unique optical properties. Gold nanoparticles (AuNPs) are considered particularly promising nanocarriers due to their excellent biocompatibility, distinctive surface plasmon resonance properties, and the relative ease with which they can be conjugated to natural compounds. In addition to being drug carriers, they enable combined therapeutic approaches, acting as photothermal agents capable of amplifying antitumor effects through synergistic mechanisms.

In preclinical studies, Yuan et al. [16] reported that fucose-grafted EGCG–AuNPs markedly improved cellular uptake in gastric cancer cell lines, resulting in significantly enhanced antitumor activity compared with free EGCG [16]. Similarly, Shukla et al. [17] showed that radioactive ^198^Au nanoparticles conjugated with EGCG could specifically target laminin receptors overexpressed in prostate tumors, thereby providing simultaneous therapeutic and imaging capabilities in vivo [17].

AuNPs have also been investigated in the field of metabolic diseases. A recent review highlighted that AuNPs loaded with polyphenols, such as resveratrol, can modulate insulin sensitivity and glucose uptake, suggesting potential applications in diabetes management [43]. Together, these findings underscore the potential of ligand-directed gold nanocarriers to selectively deliver phytochemicals to the diseased target, offering multifunctional therapeutic benefits. Beyond these specific examples, inorganic nanocarriers as a class share several overarching advantages that make them attractive for drug delivery applications.

### 3.3. Hybrid Nanoparticles for the Delivery of Natural Bioactives

Hybrid nanoparticles (NPs) are designed to combine the strengths of both organic and inorganic carriers within a single platform, thereby addressing the intrinsic weaknesses of each material class. By integrating biodegradable polymers, lipids, mesoporous silica, hydroxyapatite (HAP), or metallic nanostructures, these systems can achieve high drug loading, improved stability, controlled release, and targeted delivery of natural compounds [18,28,44,45]. At the same time, however, their structural complexity raises important challenges in terms of large-scale reproducibility and regulatory approval, which must be resolved before translation to clinical practice.

Among the different types of hybrids, solid lipid nanoparticles (SLNs) demonstrate both the potential and limitations of the approach. For instance, Rizvi et al. developed simvastatin-loaded SLNs; they had an average particle size of 144 nm, a zeta potential of −27.2 mV, and an encapsulation efficiency of 89.45%, along with excellent physicochemical stability and efficient drug entrapment [45]. While these results highlight the ability of SLNs to enhance bioavailability, they also emphasize the need for careful optimization to prevent burst release and ensure consistent performance between batches.

NLCs, a type of lipid-based nanocarrier, have shown strong potential to enhance the stability and anticancer properties of terpenoid-rich essential oils. For instance, Uchôa et al. [46] developed and functionalized NLCs loaded with copaiba oil, resulting in higher cytotoxicity against MCF-7 cells compared to free oil. This improvement is largely due to cholesterol-facilitated cellular uptake and better interaction with cell membranes [46].

Another example of this approach is the delivery of thymoquinone (TQ) using the nanocarriers listed below. Thymoquinone, a hydrophobic bioactive compound found in *Nigella sativa* seeds, has attracted increasing scientific interest due to its potential therapeutic value across a range of conditions, driven by its antioxidant and anti-inflammatory effects. Its application is increasingly widespread in functional foods, medicinal products, and beauty products [47].

TQ can be nanocarried by various systems, including polymeric nanoparticles, liposomes, phospholipid nanoformulations, SLNs, nanostructured lipid carriers (NLCs), nanoemulsions, and ethosomes. These nanocarriers are used to improve TQ’s poor aqueous solubility and bioavailability, enhance its biocompatibility, and enable site-specific delivery for various therapeutic applications, including anticancer, anti-inflammatory, and antimicrobial therapies [48,49,50].

Thymoquinone has also been shown to protect brain cells from oxidative stress, especially in regions of the brain involved in memory. TQ exhibits antineurotoxic properties, suggesting it may help prevent neurodegenerative diseases such as Alzheimer’s and Parkinson’s, due to its antioxidant and anti-inflammatory effects that protect brain cells from damage and inflammation [47].

Advancements in TQ nanoencapsulation and its controlled release mechanisms have been provided, and lipid-based, biopolymeric, and inorganic nanocarriers are particularly effective in TQ delivery.

More generally, the synergistic relationship between the organic and inorganic components allows hybrid nanocarriers to perform multiple functions simultaneously, such as protecting unstable compounds and their stimuli-triggered release. However, this multifunctionality leads to increased production complexity, with multi-step synthetic processes that can be time-consuming and require significant financial investments. Recent reviews confirm this dual nature: Andreani et al. [51], for example, have highlighted the significant therapeutic promise of hybrid nanomedicines based on natural compounds, while also highlighting persistent issues related to stability, scalability, and regulatory barriers [51].

One important subgroup of Hybrid systems, which combine polymers such as PLGA or chitosan with inorganic scaffolds like mesoporous silica nanoparticles (MSNs) or hydroxyapatite (HAP), exemplifies the potential of this approach to integrate complementary properties within a single carrier [18,44]. Yi et al. [18] demonstrated that MSN/HAP hybrids loaded with quercetin achieved drug loadings of about 28–30%, sustained release profiles, and enhanced antibacterial activity compared with bare MSNs [18]. In particular, the incorporation of HAP not only improved biocompatibility but also conferred osteogenic potential, making these hybrids particularly promising for wound healing and bone regeneration.

In addition, however, the increased structural sophistication of these systems introduces challenges for fabrication and reproducibility. Álvarez-Bermúdez et al. [28] highlighted how different morphologies, including core–shell, Janus, and raspberry architectures, can be achieved through miniemulsion and Pickering emulsion techniques [28]. While this morphological versatility permits precise control over particle size, shell thickness, and encapsulation efficiency, it adds layers of complexity that can hinder scalability. These design strategies are particularly relevant for hydrophobic polyphenols such as resveratrol and curcumin, where maintaining solubility and stability is essential but requires precise optimization to ensure batch-to-batch consistency.

Another widely explored class of nanocarriers is lipid-polymer hybrid nanoparticles (LPHNs), which combine the structural and functional advantages of two distinct classes of materials within a single carrier. Their architecture typically consists of a lipid outer shell surrounding a polymeric core: the lipid layer mimics biological membranes, facilitating cellular uptake and enhancing interactions with biological systems, while the polymeric matrix provides stability and allows a controlled release of natural compounds over time [45]. This dual design not only reduces the risk of burst release but also improves the oral bioavailability of flavonoids and supports topical administration of antioxidant compounds.

A related class of hybrid systems, cubosomes, which are nanostructured liquid crystalline particles composed of lipids and stabilizers, further demonstrates the multifunctionality of carriers. These particles offer stability and precise control over drug release, making them particularly attractive for hydrophilic anticancer agents. Saber et al. developed nano-cubosomes incorporating cisplatin alone or in combination with metformin, demonstrating significantly enhanced cytotoxicity against HCT-116 colorectal cancer cells [27]. Interestingly, the co-loaded formulation enhanced apoptosis by simultaneously modulating metabolic pathways such as AMPK/mTOR and Akt/mTOR, while also promoting oxidative stress and inhibiting lactate dehydrogenase activity. These findings highlight how polymer-lipid hybrids can be engineered not only to improve delivery efficiency but also to exploit synergistic mechanisms of action, although their structural complexity may pose challenges for reproducibility and scale-up. Taken together, these examples highlight the broader advantages of hybrid nanocarriers, which stem from their ability to blend the complementary properties of materials into multifunctional delivery platforms.

### 3.4. Stimuli-Responsive and Surface-Decorated Nanoparticles

Stimuli-responsive nanoparticles are advanced delivery systems designed to release natural bioactives in response to specific internal or external triggers, such as pH, temperature, redox environment, light, or enzymatic activity. When combined with surface decoration strategies (ligands, antibodies, peptides), these nanoparticles enable site-specific delivery and improved therapeutic efficacy, while minimizing systemic side effects [26,45,52].

Many pathological environments, such as tumor tissues or inflamed sites, exhibit a slightly acidic extracellular pH (pH 5–6) compared to the normal physiological value of 7.4. In this context, the ionization state of pH-sensitive polymers plays a crucial role. When the environmental pH falls below the polymer’s pKa, protonation of acidic or basic moieties alters chain conformation and solubility, triggering matrix swelling or degradation [53,54,55,56]. For example, polymers such as Eudragit S100 and chitosan derivatives, whose ionization behavior is governed by their pKa values, remain stable at physiological pH but become protonated and permeable in acidic environments, thus facilitating selective drug release [57]. Stimuli-responsive nanoparticles based on pH-sensitive polymers (e.g., Eudragit, chitosan derivatives) or functionalized silica can exploit this difference to selectively trigger drug release. Yi et al. [18] reported MSN/HAP hybrid particles functionalized with amino groups, which showed faster release of quercetin under acidic conditions, leading to enhanced antibacterial activity [18]. Similarly, Zhang et al. [44] described carbohydrate polymer-based nanoparticles that degrade more rapidly in acidic tumor microenvironments, thereby enhancing the intracellular accumulation of polyphenols [44]. Some pathological tissues have high intracellular levels of specific enzymes (e.g., esterases, proteases) or glutathione (GSH).

The intracellular GSH concentration in tumor cells (1–11 mM) can exceed that of the extracellular milieu (~2 µM) by several orders of magnitude. This sharp redox gradient accelerates the cleavage of disulfide linkages incorporated into polymer backbones or cross-linkers, as demonstrated in FA-CMC-GNA nanoparticles, which released nearly 80% of the payload at 5 mM GSH compared with <10% in its absence [58]. Consequently, the difference in glutathione concentration between tumor and normal tissues provides an effective biochemical trigger for selective intracellular release [58].

Polymeric nanoparticles cross-linked with disulfide bonds or enzyme-cleavable linkers enable controlled release only after cellular uptake, preventing premature degradation of sensitive natural compounds [26,27].

Gold nanoparticles (AuNPs) and other plasmonic nanostructures are capable of absorbing near-infrared (NIR) light, generating localized heat, and triggering the release of drugs. Shukla et al. [17] developed ^198^Au-EGCG nanoparticles that combine targeted radiotherapy and photothermal effects in prostate cancer models, demonstrating dual-modality therapy [17]. These strategies are particularly advantageous for solid tumors, where spatial control of drug release is crucial.

In addition to traditional pH-, redox-, and light-responsive carriers, novel magnetically activated systems have also been introduced. Ziegler et al. [59] reported an electrospun hybrid fiber incorporating magnetic nanoparticles and mesoporous silica, capable of remotely controlled release of curcumin upon external magnetic stimulation. This proof-of-concept highlights how multi-stimuli-responsive systems could broaden the applicability of natural bioactive drugs to localized therapies [59].

To stimulate responsiveness, surface modification represents a crucial strategy to enhance selective binding to target tissues. PEGylation increases stability and circulation time, reducing clearance by macrophages [3], while ligand conjugation (e.g., odoranalectin, transferrin, hyaluronic acid) allows tissue-specific targeting, such as intranasal administration to the brain [13]. Antibody-functionalized nanoparticles further enhance the accumulation of these nanoparticles in tumor cells that overexpress specific receptors. The integration of stimuli-responsiveness with ligand-mediated targeting thus offers a powerful platform for the precise delivery of phytochemicals, maximizing the therapeutic index and minimizing toxicity.

### 3.5. Biomimetic Nanocarriers: Red Blood Cell–Based Systems

Red blood cells (RBCs) and their membrane-derived extracellular vesicles (RBC-EVs) have recently emerged as promising biomimetic vectors for drug delivery, exploiting the natural components of the membrane to achieve stealth properties [60].

Their intrinsic biocompatibility, long circulation time, and ability to evade immune recognition make them attractive alternatives to synthetic nanomaterials as reported by Antonelli et al. [60,61,62]. By camouflaging drug molecules or incorporating them within the red blood cell membrane, these systems can prolong systemic residence, reduce immunogenicity, and improve therapeutic efficacy. Safarpour et al. [22] created red blood cell membrane vesicles encapsulating curcumin (RBCM-Cur vesicles), demonstrating how sonication time can influence vesicle size, zeta potential, and release kinetics, while maintaining high entrapment efficiency due to the amphiphilic nature of the red blood cell membrane [22]. Tang et al. [23] also investigated the production of red blood cell-mimicking liposomes (RC-Lips) to deliver curcumin. Their study highlights that the preparation aids diabetic wound healing by effectively modulating the inflammatory response and polarizing macrophages towards an anti-inflammatory phenotype [23].

RBC-derived extracellular vesicles (EVs) represent another category of nanosized carriers among liposomal mimetics. In the paper of Nguyen et al. [63], the RBC-EVs were deeply characterized in relation to hemolysis, highlighting their physicochemical properties and potential role as natural vectors [63]. Usman et al. [64] developed large-scale RBC-EVs capable of delivering therapeutic oligonucleotides and CRISPR components (Cas9 mRNA and guide RNAs) in both human cells and xenograft mouse models, with no observable cytotoxicity [64].

Biagiotti et al. [65] refined the production process through a “soft extrusion” method, which resulted in high yield, uniform physical properties, and efficient delivery of miR-210 to endothelial cells [65]. More broadly, extracellular vesicles have been shown to exploit cloaking and immune-evasion strategies to prolong circulation and enhance therapeutic efficacy, supporting their promise as versatile delivery platforms [66].

Joshi et al. [67] emphasized the diagnostic and therapeutic relevance of RBC-EVs, noting their function as both delivery systems and biomarkers in inflammatory and hematological disorders [67].

These studies indicate that RBC-based systems offer important benefits for the delivery of natural compounds, as their membrane-derived structure supports prolonged circulation, lowers systemic toxicity, and provides adaptable release profiles. At the same time, unresolved issues remain, particularly regarding large-scale production, sterilization, and regulatory approval. Moving toward clinical application will require standardized protocols for the isolation and characterization of RBC membranes and extracellular vesicles.

As summarized in Table 2, RBC-based nanocarriers offer unique advantages compared to synthetic systems, including intrinsic biocompatibility, immune evasion, and prolonged circulation time. Preclinical studies highlight their versatility in delivering both small hydrophobic molecules such as curcumin and large nucleic acid cargos, supporting applications that range from wound healing to gene therapy and biomarker discovery.

## 4. Targeting Strategies and Release Mechanisms for Nanoparticle-Based Delivery of Natural Bioactives

One of the main advantages of utilizing nanovectors for natural drug delivery is their ability to enhance tissue-specific accumulation and control release kinetics, thereby improving therapeutic efficacy and minimizing off-target effects and systemic toxicity. In this chapter, we provide a detailed overview of passive and active targeting approaches, stimuli-responsive release mechanisms, and their combination for precision drug delivery.

### 4.1. Passive Targeting

Passive targeting exploits the enhanced permeability and retention (EPR) effect. This phenomenon is typical of inflamed and tumorous tissues, in which permeable vascularization and altered lymphatic drainage favor the accumulation of nanocarriers [13]. Nanoparticles with dimensions generally between 50 and 200 nm can pass through these permeable vessels, remaining trapped in pathological tissues for extended periods. This mechanism does not require any specific ligand-receptor interaction and has therefore been widely used as a first-line strategy to enhance the local accumulation of bioactive compounds [26]. Several natural molecules encapsulated in PLGA nanoparticles, PEGylated micelles, or in mesoporous silica carriers have shown significantly higher intratumoral accumulation compared to their free counterparts, mainly due to prolonged circulation times and passive absorption [3,6,16]. However, it is known that the EPR effect varies considerably between patients and tumor types; This aspect may limit reliability in the clinical setting. Thus, passive targeting is increasingly being combined with nanoparticles decorated with ligands designed to actively recognize those receptors overexpressed on diseased cells.

### 4.2. Active Targeting

Active targeting strategies are based on the functionalization of the surface of nanoparticles with ligands capable of selectively binding to receptors overexpressed in tumor tissues, inflammatory sites, or endothelial cells of the central nervous system. This approach uses a broad repertoire of ligands, including peptides and lectins, including odoranalectin, which was shown to enhance intranasal delivery of curcumin to the brain [15]. Small molecules such as folic acid, transferrin, and hyaluronic acid are also commonly used to interact with folate receptors, transferrin receptors, or CD44, which are frequently overexpressed in tumor cells [27,68]. Monoclonal antibodies and synthetic aptamers also perform highly specific interactions with tumor-associated antigens. These functionalizations enhance the internalization of nanocarriers, facilitate endosomal escape, and ultimately improve the intracellular delivery of bioactive phytochemicals. Active targeting not only increases therapeutic efficacy but also reduces the required effective dose, limiting systemic exposure and potential side effects. The success of these approaches depends heavily on how drug release is regulated once the nanoparticles reach their destination, making controlled and stimuli-responsive systems a natural complement to ligand-mediated targeting.

### 4.3. Controlled and Stimuli-Responsive Release

Controlled-release strategies are designed to ensure that natural molecules are released only under specific conditions, thus maximizing therapeutic efficacy and minimizing systemic exposure. One of the most exploited mechanisms is the response to pH. Since tumor microenvironments and endosomal compartments are generally acidic (pH 5–6), many polymeric and inorganic nanocarriers were designed to destabilize or dissolve under these conditions, favoring the targeted release of drugs at the pathological site and preventing their early loss into circulation [18,44]. For example, cubosomes coated with the pH-sensitive polymer Eudragit-S100 remain stable in the upper gastrointestinal tract and selectively dissolve in the colon (pH > 7.0), promoting localized release and reducing systemic toxicity [27].

Another common approach is based on the redox environment. Nanoparticles containing disulfide bonds are able to sense high concentrations of glutathione (GSH) inside cells, a characteristic of many tumor cells. Once internalized, these nanoparticles cleave, promoting the targeted release of flavonoids and polyphenols at intracellular sites of action [26,27].

Enzyme-sensitive systems offer an additional level of control. By inserting polymer coatings or cleavage linkers sensitive to esterases, proteases, or hyaluronidases, nanoparticles can utilize upregulated enzymatic activity in diseased tissues to trigger targeted drug release [27,68].

Beyond endogenous triggers, nanocarriers can also be designed to respond to external stimuli. Gold and magnetic nanoparticles, for instance, can be activated by near-infrared (NIR) light, radiofrequency, or magnetic fields, enabling on-demand release and combination therapies such as photothermal treatment with epigallocatechin gallate (EGCG) or curcumin [17,19]. These approaches provide spatial and temporal control over drug release, enhancing both precision and efficacy.

These pH, redox, enzyme, and external trigger systems highlight the great potential of stimuli-responsive nanocarriers. However, their effect can be further enhanced when combined with active ligand-mediated targeting, resulting in multifunctional platforms that combine selective accumulation and controlled release, as discussed in the following section.

### 4.4. Combination of Targeting and Stimuli-Responsiveness

The physical characteristics of nanocarriers can also serve as cues for cellular modulation. Eichholz and Hoey demonstrated that scaffold architecture, when designed through melt electrospinning (MEW) writing, directly influences mechanotransduction. Highly aligned microfibrous scaffolds (S90 and S45) promoted increased cytoskeletal tension and nuclear localization of YAP, a central mechanosensitive transcription factor, thus guiding stem cell fate decisions more effectively than random or low-angle configurations (S10) [26]. These findings highlight how structural design can complement biochemical targeting in shaping biological responses.

Based on this principle, recent advances have focused on integrating ligand-mediated targeting with stimuli-responsive release, leading to the development of multifunctional platforms capable of site-specific accumulation, controlled drug release, and even combining therapeutic and imaging functions (theranostics). Such “smart” nanocarriers were realized in several systems, including ligand-decorated mesoporous silica nanoparticles, PLGA-based formulations, and gold nanostructures, which have shown improved therapeutic index and reduced systemic exposure in oncological and neurological models [15,17,18,19].

The intrinsic properties of tissue-delivery nanocarriers are also being exploited as targeting strategies. In one study, it was demonstrated that solid lipid nanoparticles (SLNs) can passively accumulate in the liver, providing a convenient route for delivering lipid-regulating drugs. In hyperlipidemic rats, simvastatin-loaded SLNs significantly increased hepatic drug exposure and improved pharmacodynamic outcomes without requiring active targeting ligands [45].

Therefore, combining targeting modalities with responsive release mechanisms can substantially increase the precision and efficacy of natural bioactive drug delivery. However, their translation into clinical settings remains limited by several challenges. The variability of the EPR effect across patients and tumor types limits the predictability of passive targeting. The density, orientation, and stability of ligands critically determine the performance of active targeting, and complex, oversized projects often encounter regulatory hurdles due to uncertainty about long-term safety.

New research challenges should focus on patient-specific biomarkers, scalable and reproducible ligand conjugation techniques, and multifunctional nanoplatforms with real-time imaging and on-demand release capabilities. By addressing these aspects, next-generation delivery systems can bridge the current translational gap and unlock the full therapeutic potential of natural compounds.

## 5. Preclinical Applications of Nanoparticle-Based Delivery of Natural Bioactives

Preclinical studies provide critical insights into the efficacy, pharmacokinetics (PK), biodistribution, and safety of nanoparticle formulations carrying natural bioactive compounds. This section summarizes major in vivo and advanced in vitro applications.

### 5.1. Anticancer Applications

Curcumin-loaded PLGA and PLGA-PEG nanoparticles, prepared by emulsion solvent evaporation, significantly improved the pharmacokinetic profile of curcumin, with oral bioavailability increased by 15.6- and 55.4-fold compared to free suspension and by 3.5-fold compared to PLGA alone. These improvements in C_max_, T_max_, t_1/2_, and AUC led to significant tumor growth inhibition in rodent models [3]. Inorganic carriers also showed strong antitumor potential. Fucose-carboxymethylchitosan (FU-CMC)-EGCG gold nanocomposites induced ~89% tumor cell apoptosis at concentrations up to 20 mg/L, without toxicity in HaCaT keratinocytes, and showed superior gastric antitumor activity in vivo [16]. A further advance is represented by radiolabeled ^198^Au-EGCG nanoparticles, synthesized through a biocompatible route that avoids the use of toxic reagents, which combine therapeutic and imaging functions. These theranostic nanostructures retained over 70% of the injected dose within the tumor tissue, achieved excellent antitumor efficacy in prostate cancer models, and ensured radiological safety, as the decay product (^198^Hg) remains far below EPA toxicity thresholds [17]. These results, summarized in Table 3, highlight how radiolabeled, hybrid, polymeric inorganic nanocarriers improve the bioavailability, selectivity, and multifunctionality of natural polyphenols, providing superior efficacy compared to conventional chemotherapy or radiotherapy approaches.

### 5.2. Neuroprotective Effects

Odornalectin-conjugated PEG-PLGA NPs in nasal administrations successfully delivered curcumin to the brain, improving bioavailability and reducing neuroinflammation in rodent models of neurodegenerative diseases [15,19].

Intranasal administration of PLGA nanoparticles has emerged as a promising strategy to increase the brain bioavailability of natural compounds with neuroprotective potential. This pathway bypasses first-pass metabolism and the blood–brain barrier (BBB), allowing direct transport through the olfactory and trigeminal nerves [30,31].

Preclinical studies have demonstrated that intranasal administration of PLGA nanoparticles loaded with curcumin or resveratrol results in increased CNS accumulation, reduced neuroinflammation, and improved behavioral outcomes in rodent models of neurodegenerative disorders [15,30,31]. These findings suggest that nose-to-brain nanocarrier systems may offer a clinically translatable approach for the management of Alzheimer’s disease, Parkinson’s disease, and other neurodegenerative conditions.

### 5.3. Anti-Inflammatory and Antioxidant Therapies

Quercetin-loaded MSN/HAP hybrid nanoparticles exhibited strong anti-inflammatory effects in vitro and enhanced wound healing in vivo [1,18]. Polymeric micelles containing resveratrol reduced oxidative stress signals and protected against cardiac ischemia–reperfusion injury [69,70].

Additionally, polymeric and hybrid nanoparticles, as well as biomimetic carriers, have also shown promise in inflammatory models. Tang et al. [23] developed red blood cell-mimicking liposomes (RC-Lips) encapsulating curcumin for the treatment of diabetic wounds. These vesicles combine the physicochemical stability of liposomes with the innate biocompatibility of red blood cell membranes, resulting in prolonged circulation and efficient delivery of curcumin. In vivo, RC-Lips significantly accelerated wound closure by modulating the inflammatory microenvironment, promoting macrophage polarization toward an anti-inflammatory M2 phenotype, and enhancing re-epithelialization. These findings highlight the potential of red blood cell-based biomimetic nanocarriers to extend the therapeutic potential of natural compounds beyond systemic diseases, offering new opportunities in tissue repair and regenerative medicine [23].

These reports indicate that both synthetic and biomimetic nanocarriers are capable of enhancing the anti-inflammatory and antioxidant effects of natural drugs in various pathological contexts. While polymeric and hybrid nanoparticles primarily act by improving solubility, stability, and release kinetics, red blood cell-based systems offer the additional benefit of immune evasion and prolonged circulation, resulting in greater therapeutic efficacy in chronic inflammatory conditions such as diabetic wounds. These findings broaden the therapeutic landscape of natural compounds and lay the groundwork for exploring their complementary role in antimicrobial applications, where similar pathophysiological environments and immune-modulatory mechanisms are critically involved.

### 5.4. Antimicrobial Applications

Several natural bioactives exhibit intrinsic antimicrobial properties that can be further enhanced by nanocarrier-based formulations. Nanoparticles enhance bactericidal efficacy by protecting unstable phytochemicals, prolonging their residence time, and facilitating bacterial interaction through the microbial membrane. Carriers such as silica-hydroxyapatite hybrid nanoparticles, polymeric micelles, and biomimetic RBC-derived vesicles represent promising solutions for combating infections and promoting tissue repair in complex pathological settings.

Hybrid nanoparticles loaded with quercetin and catechin showed strong bactericidal activity, achieving a 2–3 log reduction in bacterial viability compared to free compounds [18]. Amino-functionalized mesoporous silica/hydroxyapatite (MSN/HAP) hybrids loaded with quercetin achieved drug loadings of approximately 29% and exhibited pH-sensitive release, with faster liberation of quercetin in acidic environments. This feature significantly improved antibacterial efficacy, resulting in >95% inhibition of *S. aureus* and *E. coli* at 256 mg L^−1^, a performance superior to that of free quercetin. The enhanced effect was attributed to the increased bioavailability conferred by the MSN/HAP hybrid platform, which also preserved the mesoporous structure and maintained a high surface area for efficient drug delivery [18].

### 5.5. Pharmacokinetics, Biodistribution, and Safety Profiles

Nanoparticle formulations improve key pharmacokinetic parameters of phytochemicals in vivo, including maximum plasma concentration (C_max_), area under the curve (AUC), and half-life. Radiolabeled carriers demonstrate preferential accumulation in target tissues with reduced distribution to healthy organs [3,17,71].

For example, curcumin-loaded PLGA and PLGA–PEG nanoparticles extended the half-life from about 1 h to 3.9 and 6.0 h, respectively, and increased bioavailability by 15.6-fold (AUC, 8.8 to 137 µg·h/mL) and 55.4-fold (AUC, 8.8 to 486 µg·h/mL) compared to free curcumin following oral intake in rats [3]. Similarly, Xie et al. developed PLGA nanoparticles that raised the plasma half-life from 74 to 135 min and boosted relative bioavailability by 5.6-fold (AUC, 376 to 2066 µg·h/mL) [4]. In another case, PLGA–PEG–PLGA triblock copolymer micelles increased the terminal half-life and mean residence time by 4.5-fold (0.7 to 3.1 h) and 2.7-fold (1.7 to 4.6 h), respectively, while also directing biodistribution towards the lung and brain and reducing uptake by the liver and spleen [5]. Overall, these studies demonstrate how polymer architecture and PEGylation govern circulation half-life and tissue targeting.

Previously, studies have reported that gold nanoparticles of different sizes (1.4 nm and 18 nm) exhibit different translocation and accumulation profiles in biodistribution studies following their intratracheal instillation [72]. Geiser et al. reviewed the biokinetics and clearance of nanoparticles deposited in the respiratory tract. They compared nanometer-sized particles with micrometer-sized particles in the 0.5–10 μm range [73].

Similar trends were observed for other natural compounds. Abdelmonem et al. [74] reported a 7-fold increase in half-life (3.25 to 21.8 h) and a ten-fold rise in AUC (804 to 7733 µg·h/mL) for TPGS-functionalized luteolin lipid–polymer nanoparticles [74], while pH-sensitive hybrids of chitosan, sodium-alginate, and CeO_2_ extended curcumin stability and biological half-life beyond 3 h [75]. Zhang et al. [76] highlighted that multiple PEG-modified nanocarriers could achieve up to 5-fold increases in AUC and maintain prolonged systemic exposure [76].

Biomimetic carriers like RBC-derived extracellular vesicles (RBC-EVs) and RBC membrane-coated nanoparticles (RBCM-NPs) are known for their naturally long systemic circulation. Their inherent membrane proteins, especially CD47 and sialic-acid residues, help evade immune evasion by suppressing macrophage uptake, leading to significantly longer half-lives, up to 40 h, compared to 15–16 h for PEGylated versions, and lower hepatic clearance [65,77]. These platforms combine biocompatibility with effective tissue targeting and serve as important standards for evaluating synthetic nanocarriers.

Usman et al. also showed that human RBC-derived EVs preserve 40% of circulating vesicles after 6 h and 3–4% after 12 h in mice, with an apparent half-life of around 6–8 h. When administered intraperitoneally, RBC-EVs mainly accumulated in the liver, spleen, and bone marrow, with approximately 40% uptake by marrow cells, and effectively delivered RNA therapeutics without causing toxicity or weight loss. In models of leukemia and breast cancer, antisense- or Cas9-loaded RBC-EVs suppressed over 90% of oncomiRs and led to significant tumor regression, demonstrating their stability in circulation and compatibility with the immune system [64].

In contrast, unmodified extracellular vesicles (EVs) have shown a much shorter half-life of approximately 30 min in some in vivo studies using athymic nude mice, with clearance occurring within 6 h [78]. To overcome this limitation, engineered EVs, which undergo appropriate surface engineering and drug loading, are designed to enhance PK, thereby increasing stability and circulation time [79].

Another innovative approach involves a fusion system that combines extracellular vesicles (EVs) and liposomes. This EV-liposome system demonstrated a 1.33-fold increase in half-life and a 1.5-fold higher mean residence time compared to liposomes alone, along with a twofold rise in the area under the curve (AUC). A higher AUC indicates that more drug remains available to the body over time compared to liposomes alone. This hybrid system effectively merges the drug-loading capacity of liposomes with the natural targeting and low immunogenicity of EVs. Thus, it significantly increased the drug’s half-life and residence time, suggesting that the drug remains in circulation longer [80].

Future research may benefit from computer-based modeling approaches in the therapeutic development of nanocarriers. For example, physiologically-based pharmacokinetic (PBPK) modeling is a useful tool for predicting the behavior of both nanoparticles and EVs [81]. PBPK modeling can predict how EVs behave in the body, including absorption, distribution, metabolism, and excretion. This information is essential for assessing the quality, safety, and effectiveness of EV-based therapeutics. By integrating data on EV properties and physiological processes, PBPK models can enhance drug delivery system design, such as EV size and composition, administration route, and drug dosage.

While biodegradable nanocarriers are generally well-tolerated, long-term studies are still needed to evaluate chronic inflammation, immune responses, and clearance pathways, particularly for non-biodegradable systems [6,20,24]. Among current examples, the cisplatin–metformin nano-cubosome formulation exemplifies this dual advantage of efficacy and safety: in HCT-116 cells, co-loading increased cisplatin uptake by 1.5–1.6-fold and produced a synergistic cytotoxic effect (combination index = 0.606) through enhanced ATP and glucose depletion and AMPK activation. In vivo, this formulation reduced systemic toxicity by confining drug accumulation within tumor tissues and modulating mTOR/Akt, ROS, and caspase-3 pathways, thereby maximizing therapeutic efficacy while minimizing off-target damage [27].

### 5.6. Preclinical Pharmacokinetics and Lipid Disorders

Solid lipid nanoparticle (SLN) formulations have also been successfully applied to the treatment of metabolic diseases. Rizvi et al. [45] demonstrated that simvastatin-loaded SLN significantly improved systemic bioavailability compared to free simvastatin, with a threefold increase in AUC (from 259.89 to 805.72 ng h/mL) and an increase in C_max_ from 14.14 to 41.83 ng/mL. The improved pharmacokinetics were accompanied by enhanced pharmacodynamic activity, including greater reductions in total cholesterol, LDL cholesterol, and triglycerides in hyperlipidemic rats [45]. The biphasic release profile, characterized by an initial burst followed by sustained release over 72 h, further supports the use of SLN for prolonged therapeutic effects in chronic lipid disorders.

### 5.7. Stem Cell Differentiation Through Scaffold Architecture

Microarchitectural cues present in biomaterial scaffolds can direct stem cell lineage differentiation even without the use of intense biochemical stimuli. In a preclinical in vitro model, Eichholz and Hoey reported that human mesenchymal stem cells (hMSCs) cultured on MEW-fabricated scaffolds with highly ordered microfibers (S90, S45) exhibited increased expression of osteogenic markers such as alkaline phosphatase (ALP), collagen deposition, and matrix mineralization. These effects were maintained for up to 21 days in culture and were strongly associated with increased nuclear levels of YAP, suggesting that mechanical patterning of the scaffold plays a central role in directing cell fate [26,82]

## 6. Clinical Translation of Nanoparticle-Based Delivery of Natural Bioactives: Barriers and Perspectives

Despite an abundance of promising preclinical data, the clinical translation of nanoparticle-based delivery systems for natural bioactive compounds remains limited. This translational gap arises from a combination of technological, biological, regulatory, and commercial challenges that must be addressed before these advanced formulations can reach patients [21,24,71,83,84]. Nonetheless, a growing number of studies are beginning to explore clinical applicability, with curcumin-based nanodrugs representing the most advanced examples to date.

### 6.1. Regulatory and Standardization Challenges

Nanoparticle-based formulations often display complex physicochemical properties that depend on material composition, surface functionalization, size, shape, and charge. These features affect biodistribution, cellular uptake, and safety, making standardization vital but difficult [21]. Regulatory agencies (e.g., EMA, FDA) currently lack specific guidelines for approving nanocarriers containing natural bioactives, especially when the active molecules are classified as nutraceuticals or food supplements [24]. The absence of unified protocols for characterization, stability testing, and toxicity assessment further hinders the approval process.

### 6.2. Safety and Long-Term Toxicity

Although many nanocarriers (e.g., PLGA, chitosan, MSN) are generally considered biocompatible, the long-term effects of repeated or chronic exposure remain poorly explored. Major concerns include the accumulation of non-biodegradable components (e.g., gold or silica nuclei) in organs [6,17,20], potential immunogenicity due to surface ligands or residual solvents [26,27], and unintended off-target effects, including interactions with the microbiome. In this regard, PEGylated formulations deserve particular attention, as prolonged or repeated exposure has been associated with the generation of anti-PEG antibodies, hypersensitivity reactions, and accelerated clearance from the bloodstream. Moreover, the limited biodegradability of high-molecular-weight PEG may lead to tissue accumulation, highlighting the need for systematic evaluation of polymer metabolism and immunogenicity during chronic administration [33,85].

Comprehensive toxicokinetic studies and chronic toxicity models are necessary to establish robust safety profiles and inform clinical dosing strategies [20,24].

### 6.3. Manufacturing and Scalability

The reproducibility and scalability of nanoparticle synthesis remain critical challenges for clinical and commercial development. Many laboratory-scale fabrication methods (e.g., nanoprecipitation, emulsion, layer-by-layer assembly) are not easily translated into good manufacturing practice (GMP) settings [5,45]. Challenges include batch-to-batch variability in size and encapsulation efficiency [3], the use of toxic solvents or surfactants that must be completely removed, and the complexity of scaling hybrid or multifunctional systems with multiple components [28,44]. To address these issues, efforts are underway to develop continuous manufacturing platforms and microfluidic-based synthesis technologies that improve process reproducibility and control. Given the growing importance of scalability in clinical translation processes, it is essential to compare different manufacturing technologies based on efficiency, costs, and reproducibility. Table 4 summarizes the main characteristics of batch, continuous, and microfluidic manufacturing, highlighting their respective advantages, limitations, and compatibility with carriers for nanomedicine applications.

### 6.4. Clinical Design and Biomarker Selection

Designing clinical trials for nanoparticles loaded with natural drugs requires careful attention to patient selection, endpoints, and biomarkers. Many natural compounds operate via pleiotropic mechanisms, making it difficult to define specific outcome measures [1,70]. Furthermore, the variability of the EPR effect across different tumor types and patients limits the predictive power of preclinical models [13,71]. Personalized strategies, such as the use of biomarkers to define inclusion criteria or imaging-guided administration, may increase the chances of successful clinical translation.

### 6.5. Intellectual Property and Commercialization

Because many natural compounds are not patentable, pharmaceutical companies have limited financial incentives to invest in expensive nanodelivery technologies. Innovation, therefore, tends to focus on proprietary nanocarrier systems (e.g., hybrid structures, ligand-decorated particles) [18,45] combinatorial formulations of natural and synthetic molecules, and companion diagnostics to enable targeted and personalized therapies. A supportive regulatory and commercial ecosystem is crucial for fostering academia-industry partnerships and technology transfer.

### 6.6. Emerging Clinical Evidence and Future Perspectives

Although still limited, recent studies demonstrate the translational potential of nanocarriers. Curcumin liposomes were shown to reduce inflammatory osteolysis in post-arthroplasty models, supporting applications beyond oncology [89]. A systematic review confirmed that nanoformulations improve curcumin bioavailability but also highlighted persistent barriers, such as variable patient responses [90]. Other authors stressed the need for advanced carriers, including nanoparticles and hydrogels, to enhance efficacy and minimize drug–drug interactions [91]. The ongoing NCT05768919 Phase I/II trial is evaluating intravenous liposomal curcumin (LipoCurc™,SignPath Pharma, Inc., Sandy, UT, USA) in combination with radiotherapy and temozolomide for newly diagnosed high-grade gliomas. The study employs a TITE-BOIN dose-escalation design to determine the maximum tolerated dose (240–400 mg/m^2^) and recommend the Phase II dose, with feasibility and exploratory efficacy (PFS, OS) as secondary endpoints. As of January 2025, fourteen patients had been enrolled in the dose-escalation phase. Early Phase I reports indicated good tolerability and preliminary antitumor activity [92]. These translational and clinical studies are compared in Table 5.

To translate these advances into practice, several steps are needed: harmonized regulatory frameworks for nanoformulated phytotherapeutics [24]; adoption of predictive preclinical platforms such as 3D cultures and organ-on-chip systems [21]; integration of theranostic tools for real-time monitoring [17,19] and stronger collaboration between materials science, pharmacology, clinical research, and industry. With sustained innovation and coordinated efforts, nanocarrier-based delivery of natural bioactives may progress from preclinical promise to clinical application.

## 7. Conclusions

Nanoparticle-based delivery systems represent a promising frontier in the pharmacological valorization of natural bioactive compounds, addressing long-standing issues such as poor solubility, instability, low bioavailability, and rapid metabolism, which limit their clinical potential [2,3,4]. Over the past decade, significant progress has been made in the design and optimization of nanocarriers, ranging from biodegradable polymeric systems and inorganic scaffolds to hybrid and stimuli-responsive platforms, demonstrating enhanced pharmacokinetics, tissue targeting, and therapeutic efficacy in preclinical studies [3,6,18,26,93].

In parallel, biomimetic approaches such as red blood cell-derived vesicles have recently emerged as promising alternatives to synthetic nanocarriers, offering prolonged circulation, immune evasion, and versatile drug-loading capabilities [22,23,65]. Advanced delivery strategies, including ligand-mediated targeting, enzyme- and pH-sensitive release, and theranostic integration, are enabling the precision delivery of therapeutic agents, including phytochemicals and repurposed drugs such as metformin, to diseased tissues while reducing systemic toxicity [17,19,27]. However, despite this technological maturity, clinical translation remains limited, with few formulations advancing beyond Phase I-II trials [24,71].

The main barriers focus on five critical areas: standardization and regulatory framework, long-term safety, manufacturing scalability, validity of preclinical models, and intellectual property constraints.

The primary concern is the absence of unified guidelines. Nanomaterials’ physicochemical properties, such as size, surface charge, shape, and coatings, significantly influence their biodistribution and safety; however, regulatory approaches are disorganized. The FDA in the U.S. provides guidance on nanomaterials’ characterization, quality, and stability. In Europe, the EMA has documents on issues like surface coatings, but lacks a comprehensive nanomedicine framework. Consequently, developing harmonized characterization methods (CQAs), standardized stability protocols, and shared taxonomies is crucial to enhance comparability and reproducibility [94,95].

The second obstacle involves long-term safety and biocompatibility. While biodegradable matrices like PLGA generally have a good safety profile, more detailed research is needed on the effects of chronic exposure, immune responses, and how non-degradable materials, such as gold or silica cores, are cleared from the body. Conducting toxicokinetic and chronotoxicologic studies in appropriate species, along with in vivo imaging and surrogate biomarkers, will be crucial for establishing safe dosage protocols [25].

The third concerns large-scale industrial production and challenges in scaling up the production of reproducible and GMP-compliant nanocarriers [21,24,96]. Laboratory procedures, such as emulsification, nanoprecipitation, or hybrid multicomponent approaches, often exhibit variability between batches and dependence on solvents or surfactants that need to be completely removed. The adoption of continuous manufacturing and microfluidic strategies, integrated with process analytical technology (PAT), can improve reproducibility, reduce size variability, and optimize encapsulation efficiency [25].

Furthermore, the pleiotropic nature of natural compounds complicates the design of clinical studies and the choice of endpoints [1,70].

Another limitation is represented by the low predictability of preclinical models, due to biological heterogeneity and interindividual variations in the EPR effect. To overcome these issues, adopting more translational models, such as three-dimensional cultures and organ-on-chip systems, is necessary to better mimic human physiology [1]. These models should integrate functional biomarkers, perfusion, and permeability parameters to improve the correlation between experimental response and clinical outcome [1,25].

Finally, issues related to intellectual property and industrial constraints pose another obstacle. When the natural active ingredient is difficult to patent, the focus shifts to the proprietary design of the carriers, materials, architectures, or ligands, which must demonstrate concrete and measurable clinical advantages to justify the development costs [25].

In conclusion, although delivering natural compounds via nanocarriers remains an evolving field, the intersection of nanotechnology, pharmacology, and systems biology is paving the way for safer, more effective, and clinically relevant formulations. With proper translational strategies, adopting standardized characterization protocols, such as size distribution, surface charge, encapsulation efficiency, and release kinetics, and fostering close collaboration among academia, industry, and regulatory agencies will help bridge the gap between preclinical promise and therapeutic application, and personalized medicine strategies [68].

Only through a combination of scientific rigor and practical implementation can the sector fully unlock the potential of nanotechnologies to enhance natural compounds.

## Figures and Tables

**Table 1:** Proportion of curcumin studies employing nanocarriers across different research stages (2020–2025). Values indicate the percentage of nanoformulations relative to the total number of publications in each category (LAB, ANIMAL, CLINICAL). Data were obtained from PubMed queries described in Section 2.

Year	LAB_NANO/ LAB (%)	ANIMAL_NANO/ ANIMAL (%)	CLINICAL_NANO/ CLINICAL (%)
2020	28.7	37.2	18.8
2021	28.3	28.2	9.5
2022	27.3	31.3	15.4
2023	28.8	29.0	20.0
2024	31.0	35.0	20.0
2025	31.9	30.1	7.1

**Table 2:** Advantages and preclinical applications of RBC-based biomimetic nanocarriers for natural compounds.

System	Natural Compound (s)	Main Findings	Preclinical Application	Reference
RBCM vesicles (RBCM-Cur)	Curcumin	High entrapment efficiency; tunable size, zeta potential, and release via sonication.	Potential systemic anti-inflammatory therapy	[22]
RBC-mimicking liposomes (RC-Lips)	Curcumin	Prolonged circulation; macrophage polarization to M2 phenotype; accelerated wound closure.	Diabetic wound healing, tissue repair	[23]
RBC-derived extracellular vesicles	Cas9 mRNA, gRNAs, oligonucleotides	Efficient nucleic acid delivery in human cells and xenograft mouse models; no cytotoxicity.	Gene editing and RNA-based therapeutics	[64]
RBC-EVs (soft extrusion method)	miR-210	Homogeneous vesicles; efficient delivery to endothelial cells; improved angiogenic activity.	Regenerative medicine, vascular repair	[65]
RBC-EVs	—	Highlighted role as carriers and diagnostic biomarkers in inflammatory/hematological disorders.	Biomarker discovery, theranostic potential	[67]

**Table 3:** Anticancer applications of natural polyphenol-loaded nanoparticles.

Nanocarrier/Formulation	Natural Compound	Experimental Model	Key Outcomes	Reference
PLGA and PLGA–PEG nanoparticles (emulsion solvent evaporation)	Curcumin	Rodent models (oral administration)	↑ Bioavailability 15.6-fold (PLGA) and 55.4-fold (PLGA–PEG) vs. free suspension; ↑ C_max_, T_max_, t_1/2_, AUC; ↓ clearance; significant tumor growth inhibition	[3]
FU–CMC–EGCG gold nanocomposites	Epigallocatechin gallate (EGCG)	Gastric cancer cells; in vivo gastric cancer model	~89% tumor cell apoptosis at 20 mg/L; selective effect (no toxicity in HaCaT cells); superior anticancer activity in vivo	[16]
^198^Au–EGCG radioactive gold nanoparticles	Epigallocatechin gallate (EGCG)	Prostate cancer models (theranostic approach)	>70% retention in tumor; prolonged survival; dual imaging + therapy; biocompatible synthesis; safe Hg-198 decay product (<1000-fold below EPA threshold)	[17]

Upward (↑) and downward (↓) arrows indicate an increase or a decrease of the corresponding parameter, respectively.

**Table 4:** Comparison of various large-scale production technologies.

Technology	Efficiency/ Yield/ Throughput	Initial Cost/ Investment	Product Uniformity/ Reproducibility	Compatible Carrier Types/ Application Notes
Batch Manufactory	Moderate, but with limited scalability; difficulty scaling linearly with volume (each batch requires adjustment)	Relatively low for small systems; costs increase with scale.	Batch-to-batch variability, wider distributions	Lipids (liposomes, SLN), polymers, emulsions; often used in industrial laboratories [86].
Continuous manufacturing	High productivity, continuous integrated process	High initial investment, maintenance costs	Better uniformity, tighter control of process parameters	Polymeric nanoparticles, LNPs for mRNA therapy; e.g., VandenBerg et al. [87].
Microfluidics/ continuous flow on a microscopic scale	Low absolute productivity per channel, but potential for parallelization	Medium–high (sophisticated devices, microchip, precise pumps)	Excellent dimensional uniformity, low PDI, reproducible	Lipid, polymeric particles, hybrid nanocarriers; e.g., Gimondi et al. [88].

**Table 5:** Selected translational and clinical studies on nanoformulated natural compounds.

Compound/Formulation	Nanocarrier Type	Clinical/Translational Setting	Key Outcomes	Reference
Curcumin liposomes	Liposomal formulation	Post-arthroplasty inflammatory osteolysis (preclinical/early translational)	Reduced osteolysis, improved anti-inflammatory response	[89]
Curcumin formulations	Nanoparticles, micelles, liposomes (review)	Multiple indications (oncology, inflammation, metabolic diseases)	Improved bioavailability; identified translational barriers (heterogeneous response, limited endpoints)	[90]
Curcumin delivery systems	Nanoparticles, hydrogels	General clinical use (narrative review)	Highlighted therapeutic potential, need for advanced delivery, and awareness of drug–drug interactions	[91]
Liposomal Curcumin (LipoCurc™)	Liposomal nanoparticle	High-grade gliomas (Phase I/II trial, NCT05768919)	Dose-escalation with RT + TMZ; endpoints: MTD, RP2D, safety; preliminary results show tolerability and early efficacy	[92]

## Data Availability

Data sharing is not applicable.

## Supplementary Materials

The following supporting information can be downloaded at: https://www.mdpi.com/article/10.3390/ma18214960/s1. File S1: pubmed_trends.py-Python 3.12 script and complete PubMed queries used to retrieve yearly publication counts (2020–2025) and classify curcumin studies by research stage (in vitro, animal, clinical) and nanocarrier use.

## Abbreviations

The following abbreviations are used in this manuscript:

ABC	Accelerated blood clearance
AKT	v-Akt murine thymoma viral oncogene homolog
ALP	Alkaline phosphatase
AMPK	AMP-activated protein kinase
AuNPs	Gold nanoparticles
BBB	Blood–brain barrier
BOIN (TITE-BOIN)	Time-to-event Bayesian Optimal Interval (dose-escalation design)
C_max_	Maximum plasma concentration
CMC	Carboxymethyl chitosan
CNS	Central nervous system
CRISPR	Clustered Regularly Interspaced Short Palindromic Repeats
EGCG	Epigallocatechin gallate
EMA	European Medicines Agency
EPR	Enhanced permeability and retention
EVs	Extracellular vesicles
FDA	Food and Drug Administration
FA	Folic acid
GNA	Gambogenic acid
GMP	Good manufacturing practice
GSH	Glutathione
HAP	Hydroxyapatite
LNPs	Lipid nanoparticles (for nucleic-acid delivery)
LPHNs	Lipid–polymer hybrid nanoparticles
mRNA	Messenger RNA
mTOR	Mechanistic target of rapamycin
MTD	Maximum tolerated dose
MEW	Melt electrospinning writing
MSNs	Mesoporous silica nanoparticles
NIR	Near-infrared
NLCs	Nanostructured lipid carriers
OS	Overall survival
PCL	Poly(ε-caprolactone)
PBLG	Poly(γ-benzyl-L-glutamate)
PBPK	Physiologically based pharmacokinetic
PEG	Polyethylene glycol
PHPMA	Poly(hydroxypropyl methacrylamide)
PDI	Polydispersity index
PD	Pharmacodynamics
PK	Pharmacokinetics
PLGA	Poly(lactic-co-glycolic acid)
PFS	Progression-free survival
ROS	Reactive oxygen species
RBC	Red blood cell
RBCM-NPs	Red blood cell membrane–coated nanoparticles
RBC-EVs	Red blood cell–derived extracellular vesicles
RC-Lips	Red blood cell–mimicking liposomes
RP2D	Recommended Phase II dose
RT	Radiotherapy
TMZ	Temozolomide
TPGS	d-α-Tocopheryl polyethylene glycol 1000 succinate
SLN/SLNs	Solid lipid nanoparticle(s)
t½ (t1/2)	Terminal half-life
T_max_	Time to C_max_
YAP	Yes-associated protein
